# Family with sequence similarity 13C (FAM13C) overexpression is an independent prognostic marker in prostate cancer

**DOI:** 10.18632/oncotarget.16357

**Published:** 2017-03-18

**Authors:** Christoph Burdelski, Laura Borcherding, Martina Kluth, Claudia Hube-Magg, Nathaniel Melling, Ronald Simon, Christina Möller-Koop, Philipp Weigand, Sarah Minner, Alexander Haese, Hans Uwe Michl, Maria Christina Tsourlakis, Frank Jacobsen, Andrea Hinsch, Corinna Wittmer, Patrick Lebok, Stefan Steurer, Jakob R Izbicki, Guido Sauter, Till Krech, Franziska Büscheck, Till Clauditz, Thorsten Schlomm, Waldemar Wilczak

**Affiliations:** ^1^ Institute of Pathology, University Medical Center Hamburg-Eppendorf, Hamburg, Germany; ^2^ General, Visceral and Thoracic Surgery Department and Clinic, University Medical Center Hamburg-Eppendorf, Hamburg, Germany; ^3^ Martini-Clinic, Prostate Cancer Center, University Medical Center Hamburg- Eppendorf, Hamburg, Germany; ^4^ Department of Urology, Section for Translational Prostate Cancer Research, University Medical Center Hamburg-Eppendorf, Hamburg, Germany

**Keywords:** prostate cancer, prognosis, immunohistochemistry, FAM13C

## Abstract

FAM13C, a gene with unknown function is included in several mRNA signatures for prostate cancer aggressiveness. To understand the impact of FAM13C on prognosis and its relationship to molecularly defined subsets, we analyzed FAM13C expression by immunohistochemistry on a tissue microarray containing 12,400 prostate cancer specimens. Results were compared to phenotype, ERG status, genomic deletions of 3p, 5q, 6q and *PTEN*, and biochemical recurrence. FAM13C was detectable in cell nuclei of cancerous and non-neoplastic prostate cells. 67.5% of 9,633 interpretable cancers showed FAM13C expression: strong in 28.3%, moderate in 24.6% and weak in 14.6%. Strong FAM13C expression was linked to advanced pT stage, high Gleason grade, positive lymph node status, and early biochemical recurrence (*p* < 0.0001 each). FAM13C expression was associated with *TMPRSS2:ERG* fusions. It was present in 85% of ERG positive but in only 54% of ERG negative cancers (*p* < 0.0001), and in 91.1% of *PTEN* deleted but in only 69.2% of *PTEN* non-deleted cancers (*p* < 0.0001). The prognostic role of FAM13C expression was independent of classical and quantitative Gleason grade, pT stage, pN stage, surgical margin status and preoperative PSA. In conclusion, the results of our study demonstrate that expression of FAM13C is an independent prognostic marker in prostate cancer. Finding FAM13C also in non-neoplastic prostate tissues highlights the importance of properly selecting cancer-rich areas for RNA-based FAM13C expression analysis.

## INTRODUCTION

Prostate cancer is the most prevalent cancer in men in Western society [1]. Although the majority of prostate cancers behave in an indolent manner, a small subset is highly aggressive and requires extensive treatment [2, 3]. Established preoperative prognostic parameters are limited to Gleason grade and tumor extent in biopsies, prostate-specific antigen (PSA), and clinical stage. Although these data are statistically powerful, they are often insufficient for optimal individual treatment decisions. It is hoped that a better understanding of disease biology will eventually lead to the identification of clinically applicable molecular markers that enable a more reliable prediction of prostate cancer aggressiveness.

FAM13C (Family with sequence similarity 13, Member C) is one of currently 857 known members of the FAM protein family. The function and the cellular localization of FAM13C-and most other FAMs-is largely unknown. Based on sequence analyses indicating the presence of a Rho GTPase-activating protein domain in exons 2–5, FAM13-proteins may be involved in intracellular signal transduction pathways relevant for cancer [4].

In prostate cancer, FAM13C has gained interest because it is–despite of its unknown function-part of several RNA expression signatures for estimating prostate cancer aggressiveness [5, 6], one of which has become commercially available [6]. The recent availability of a FAM13C specific antibody facilitates large-scale *in-situ* analysis in order to clarify whether also FAM13C protein expression can serve as a prognostic marker in prostate cancer. Such studies aiming in a systematic analysis of the prognostic value of FAM13C protein expression or its association to cancer phenotype and other molecular features of the disease are lacking. We took advantage of our large prostate cancer prognosis tissue microarray to study FAM13C expression in more than 12,000 individual prostate cancers with pathological and clinical follow-up information.

## RESULTS

### Technical issues

A total of 9,633 (77.5%) of tumor samples were interpretable in our TMA analysis. Reason for non-informative cases (2,794 spots; 22.5%) included lack of tissue samples or absence of unequivocal cancer tissue in the TMA spot.

### Prognostic impact of classical parameters

**Figure 1:**
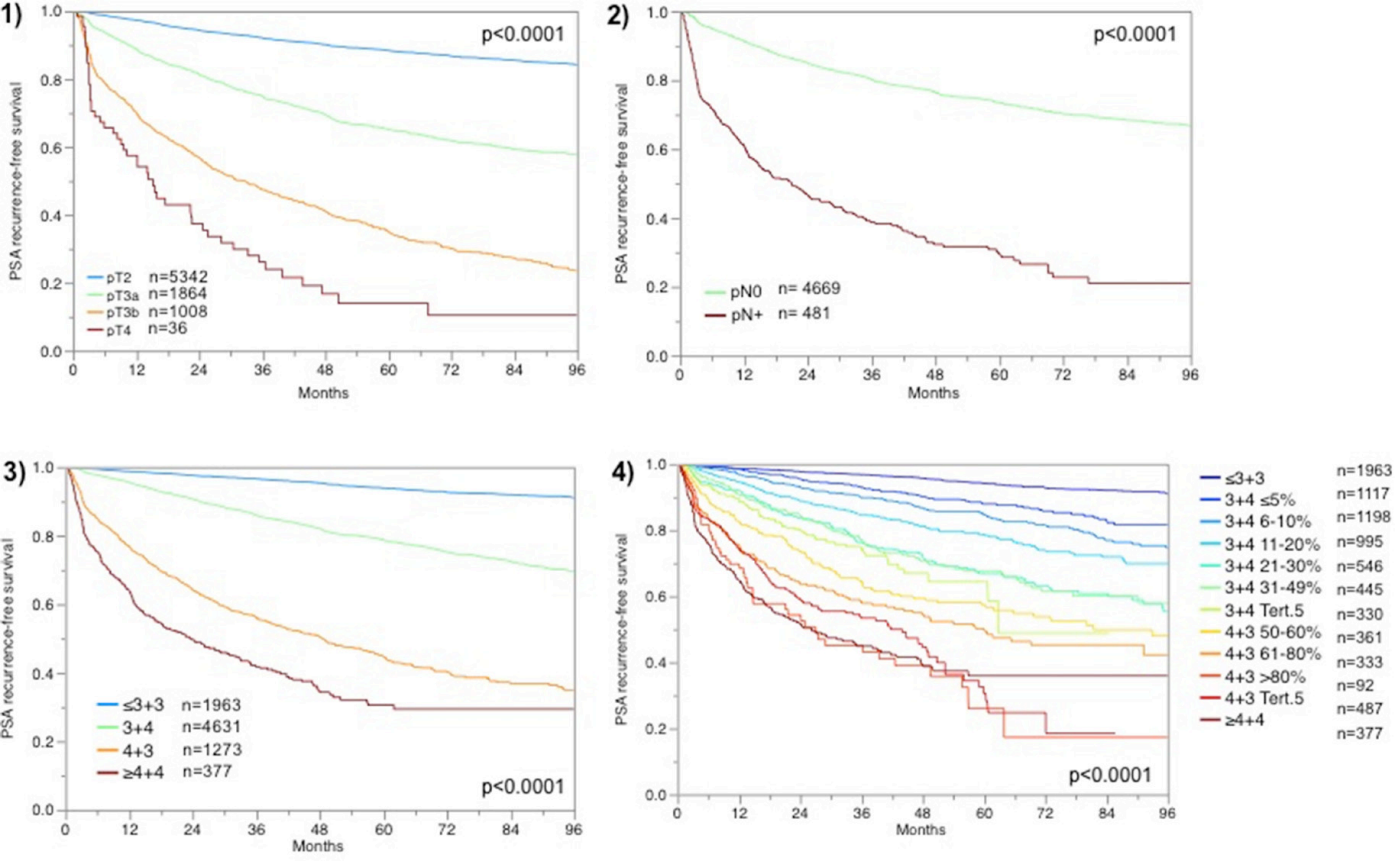
Prognostic impact of (**1**) tumor stage (pT), (**2**) lymph node stage (pN), (**3**) classical Gleason grading and (**4**) quantitative Gleason grading.

For all patients for which FAM13C immunostaining was interpretable and follow-up data were available, the prognostic role with respect to PSA recurrence is depicted in Figure 1 for pT category (Figure 1.1), pN category (Figure 1.2), classical Gleason grading (Figure 1.3) and quantitative Gleason grading (Figure 1.4). These findings indirectly validate our morphological and clinical data.

### FAM13C immunohistochemistry

**Figure 2:**
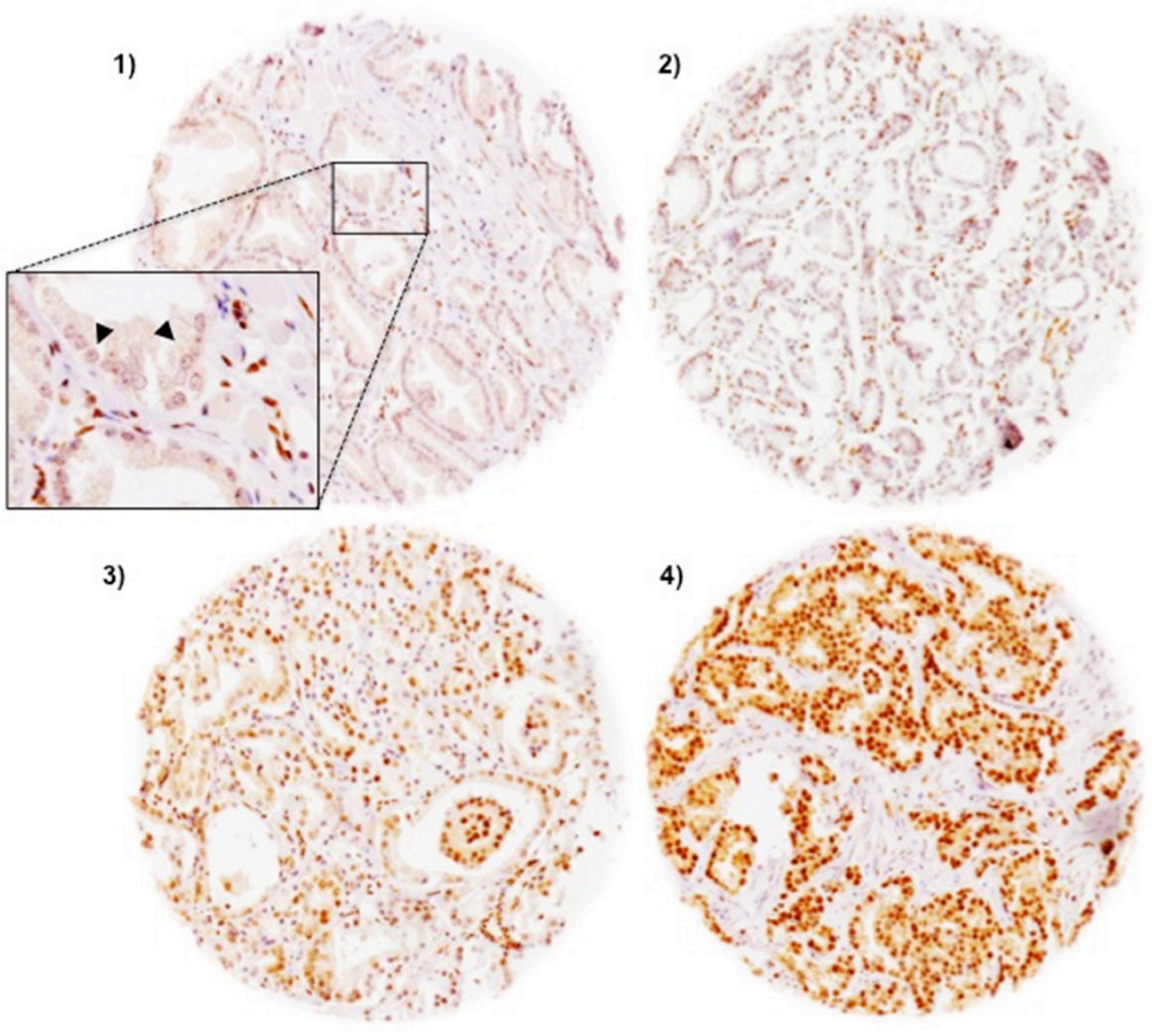
Representative pictures of FAM13C immunostaining in prostate cancer (100×) (**1**) negative, (**2**) weak (**3**) moderate (**4**) strong staining. The inset in 1) shows a magnification of FAM13C-negative cancer cells (arrowhead) and FAM13C-positive stroma cells (400×).

**Figure 3:**
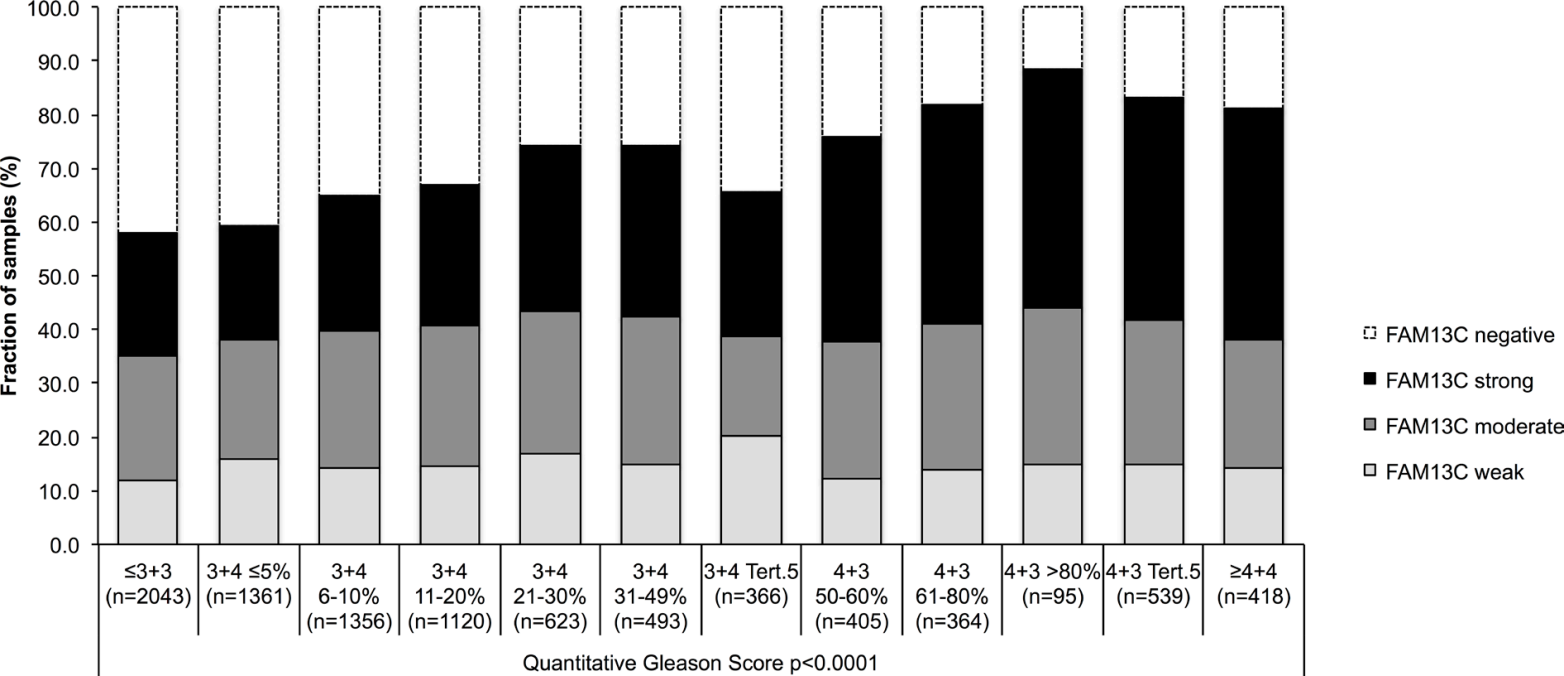
Association between FAM13C expression and the quantitative Gleason score (*p* < 0.0001) in 9,183 prostate cancers

FAM13C immunostaining was localized in the nuclei of prostate epithelial cells and usually also in stroma cells as well as lymphocytes. Staining was typically stronger in cancer cells as compared to the weak to moderate immunostaining found in basal and luminal cells of normal appearing prostate epithelium. In cancer cells, positive FAM13C immunostaining was seen in 67.5% of our 9,633 interpretable tissues and was considered weak in 14.6%, moderate in 24.6% and strong in 28.3% of tumors. Representative images of FAM13C immunostainings are shown in Figure 2. Presence of intensive FAM13C immunostaining was strongly linked to advanced pT stage, high Gleason grade, positive lymph nodes, high preoperative serum PSA, and positive surgical margin status (*p* < 0.0001 each; Table 1). Comparison with quantitative Gleason grades revealed a continuous increase of FAM13C staining with the percentage of Gleason 4 and presence of a tertiary Gleason 5 grade (*p* < 0,0001; Figure 3). To further extend our data on the relationship between FAM13C expression and different stages of benign and neoplastic prostate lesions, we analyzed a small “prostate cancer progression” TMA. This analysis revealed a continuous increase of the fraction of lesions with strong FAM13C expression from BPH (1.4%) to PIN (4.8%), high grade Gleason cancers (4.9%), nodal metastasis (26.3%) to hormone refractory cancers (37.5%). The overall higher fraction of cases with strong FAM13C expression in this TMA as compared to the large TMA is due to the fact that these TMAs were analyzed at different days using different batches of the FAM13C antibody.

**Table 1:** Association between FAM13C immunostaining results and prostate cancer phenotype

Parameter	*n* evaluable	FAM13C (%)				*p* value
		negative	weak	moderate	strong	
**All cancers**	9,633	32.5	14.6	24.6	28.3	
**Tumor stage**						*< 0.0001*
pT2	6,145	37.5	14.6	23.7	24.2	
pT3a	2,194	25.7	15.3	25.4	33.6	
pT3b-4	1,257	20.3	13.1	27.7	38.9	
**Gleason grade**						*< 0.0001*
≤ 3 + 3	2,125	42.2	12.1	23.2	22.5	
3 + 4	5,474	33.7	15.6	24.6	26.0	
4 + 3	1,520	19.3	14.1	27.0	39.5	
≥ 4 + 4	468	17.9	14.7	23.1	44.2	
**Lymph node metastasis**						*< 0.0001*
N0	5,542	31.1	15.6	24.6	28.7	
N+	575	19.1	12.3	26.3	42.3	
**Preop. PSA level (ng/ml)**						*0.001*
< 4	1,172	28.1	14.0	24.4	33.5	
4–10	5,727	33.5	14.5	24.8	27.2	
10–20	1,948	33.3	14.9	24.3	27.6	
> 20	683	30.0	14.9	23.6	31.5	
**Surgical margin**						*< 0.0001*
negative	7,617	33.7	14.6	24.4	27.2	
positive	1,836	27.9	14.3	25.3	32.5	

### Association with *TMPRSS2*:*ERG* fusion status and ERG protein expression

**Figure 4:**
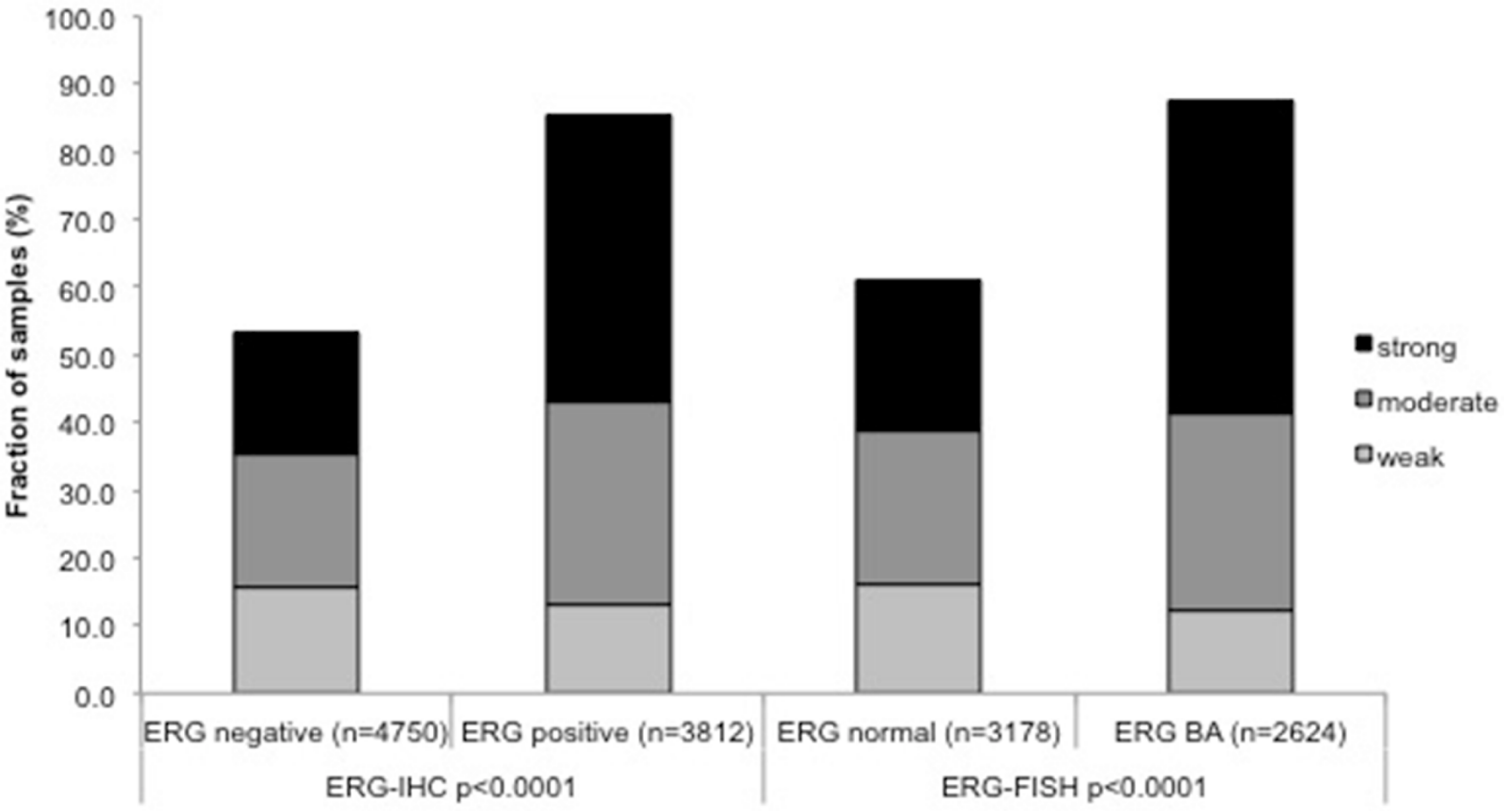
Associations between positive FAM13C immunostaining and ERG status (IHC/FISH)

To evaluate whether FAM13C staining is associated with ERG status in prostate cancers, we compared the FAM13C results with data from previous studies on our TMA (expanded from [7, 8]). Data on *TMPRSS2:ERG* fusion status obtained by FISH were available from 7,099 patients and by immunohistochemistry from 10,678 patients. Data on both ERG FISH and IHC were available from 6,778 cancers, and an identical result (ERG IHC positive and break by FISH) was found in 6,463 of 6,778 (95.4%) cancers. FAM13C expression was massively linked to the presence of ERG expression and rearrangement. FAM13C expression was found in 85.4% of cancers with immunohistochemical ERG expression and in 87.6% of tumors with *ERG* rearrangement by FISH, but in only 53.6% (IHC) and 61% (FISH) ERG negative cancers (*p* < 0.0001 each, Figure 4). FAM13C immunostaining was similarly linked to unfavorable tumor features in subsets of both ERG negative and ERG positive cancers (Supplementary Tables 1 and 2).

**Table 2:** Association between FAM13C expression and Ki67-labeling index in all prostate cancers and subsets defined by Gleason score, ERG fusion, and PTEN deletion status

	FAM13C IHC	*n*	Ki67 Li (mean)	Sth.deviation
all *p < 0.0001*	negative	1,991	2.1	0.06
	weak	837	2.8	0.09
	moderate	1,368	2.9	0.07
	strong	1,615	3.6	0.07
pGleason ≤ 3 + 3 *p < 0.0001*	negative	549	1.7	0.09
	weak	158	2.5	0.16
	moderate	288	2.5	0.12
	strong	298	2.7	0.12
pGleason 3 + 4 *p < 0.0001*	negative	1,182	2.1	0.07
	weak	520	2.8	0.10
	moderate	801	2.9	0.08
	strong	887	3.4	0.08
pGleason 4 + 3 *p < 0.0001*	negative	198	2.7	0.25
	weak	121	2.9	0.32
	moderate	219	3.6	0.24
	strong	311	4.2	0.20
pGleason ≥ 4 + 4 *p = 0.0143*	negative	53	3.4	0.60
	weak	34	3.6	0.75
	moderate	52	4.4	0.61
	strong	109	5.5	0.42
PTEN normal *p < 0.0001*	negative	955	2.5	0.09
	weak	453	3.0	0.13
	moderate	711	3.1	0.10
	strong	902	3.6	0.09
PTEN deleted *p = 0.0002*	negative	69	2.9	0.35
	weak	76	3.3	0.34
	moderate	187	3.3	0.21
	strong	328	4.2	0.16
ERG negative *p < 0.0001*	negative	1,537	1.9	0.07
	weak	464	3.0	0.13
	moderate	589	3.2	0.11
	strong	531	3.9	0.12
ERG positive *p < 0.0001*	negative	424	2.5	0.12
	weak	357	2.6	0.13
	moderate	747	2.8	0.09
	strong	1,060	3.4	0.08

### Associations with other key genomic alterations of prostate cancer

Earlier studies had provided evidence for distinct molecular subgroups of prostate cancers defined by *TMPRSS2:ERG* fusions and several genomic deletions. Others and us had previously described a strong link between *PTEN* and 3p13 deletions and ERG positivity as well as between 5q21 and 6q15 deletions and ERG negativity [9–16]. To study whether FAM13C expression might be particularly linked to a cancer subtype defined by one of these genomic deletions, FAM13C data were compared to preexisting findings on 10q23 (*PTEN)*, 3p13 (*FOXP1)*, 6q15 (*MAP3K7*) and 5q21 (*CHD1*) deletions (Figure 5.1, 5.2, 5.3). Strong FAM13C expression was significantly linked to deletions of *PTEN* and 3p13 (*FOXP1*) if all cancers were jointly analyzed (*p* < 0.0001 each). A separate analysis of ERG negative and ERG positive cancers, however, revealed that in both subgroups strong associations were limited to FAM13C and deletions of *PTEN* (*p* < 0.0001). In ERG negative cancers, FAM13C expression was also linked to deletions of 5q and 6q (*p* < 0.0001 each), although to a lesser extent as compared to *PTEN* deletions.

**Figure 5:**
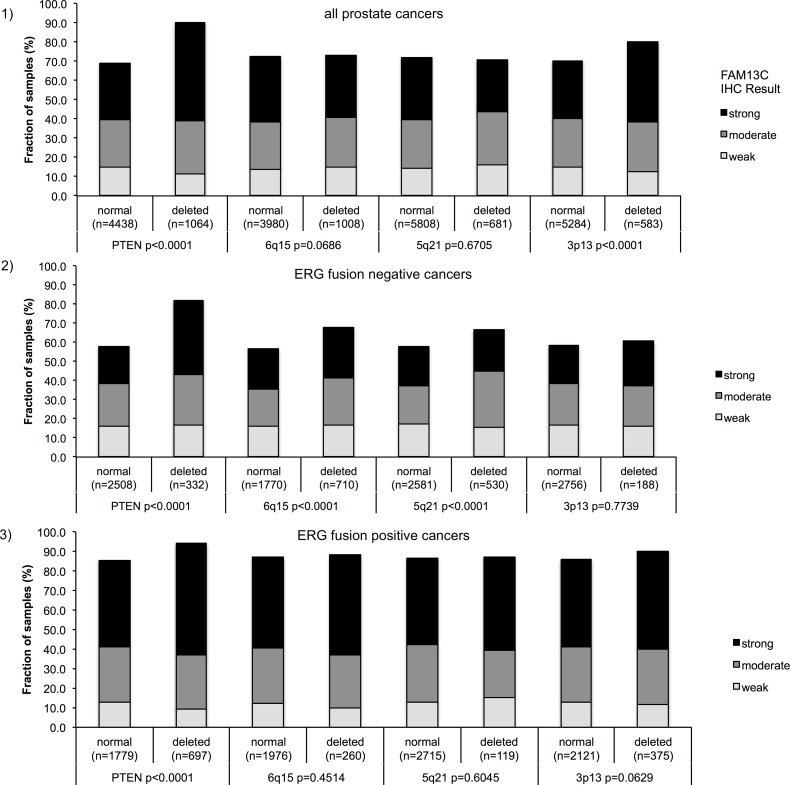
Associations between positive FAM13C immunostaining and *PTEN, 5q21 (CHD1), 6q15 (MAP3K7), 3p13 (FOXP1)* – deletion status in (**1**) all cancers, in (**2**) ERG fusion negative cancers and (**3**) ERG fusion positive cancer.

### Association with tumor cell proliferation (Ki67LI)

High levels of FAM13C staining were significantly linked to increased tumor cell proliferation (*p* < 0.0001). This association held also true with high significance (*p* < 0.0001) in most subgroups of cancers with identical Gleason grade (≤ 3 + 3; 3 + 4; 4 + 3; ≥ 4 + 4;), and was independent of the ERG status (*p* < 0.0001), or presence of *PTEN* deletions (*p* = 0.0002) (Table 2).

### Associations with PSA recurrence

Follow-up data were available from 8,675 patients with interpretable FAM13C immunohistochemistry results on the TMA. There was a significant association between strong FAM13C staining and early PSA recurrence if all tumors were jointly analyzed (*p* < 0.0001; 6.1), and also if the subgroups of ERG negative (*p* < 0.0001; Figure 6.2) and ERG positive (*p* < 0.0001; Figure 6.3) cancers were analyzed separately. FAM13C did not provide additional prognostic impact if the cancers were grouped according to the classical Gleason score (Figure 7.1). Despite a strong tendency towards a worse outcome in tumors with a high FAM13C expression in several subgroups defined by comparable quantitative Gleason grades, statistically significant differences were also not seen in these subgroups (Figures 7.2, 7.3, 7.4, 7.5, 7.6, 7.7, 7.8, 7.9, 7.10). Because of the strong link between FAM13C expression and *PTEN* deletion, the analyses was extended to tumor subgroups stratified according to the FAM13C/*PTEN* status. These analyses revealed that the prognostic impact of FAM13C expression was strong in cancers lacking *PTEN* deletions (*p* < 0.0001 Figure 6.4), and was still statistically significant in the smaller subgroup of tumors harboring *PTEN* deletions (*p* = 0.0212, Figure 6.5).

**Figure 6:**
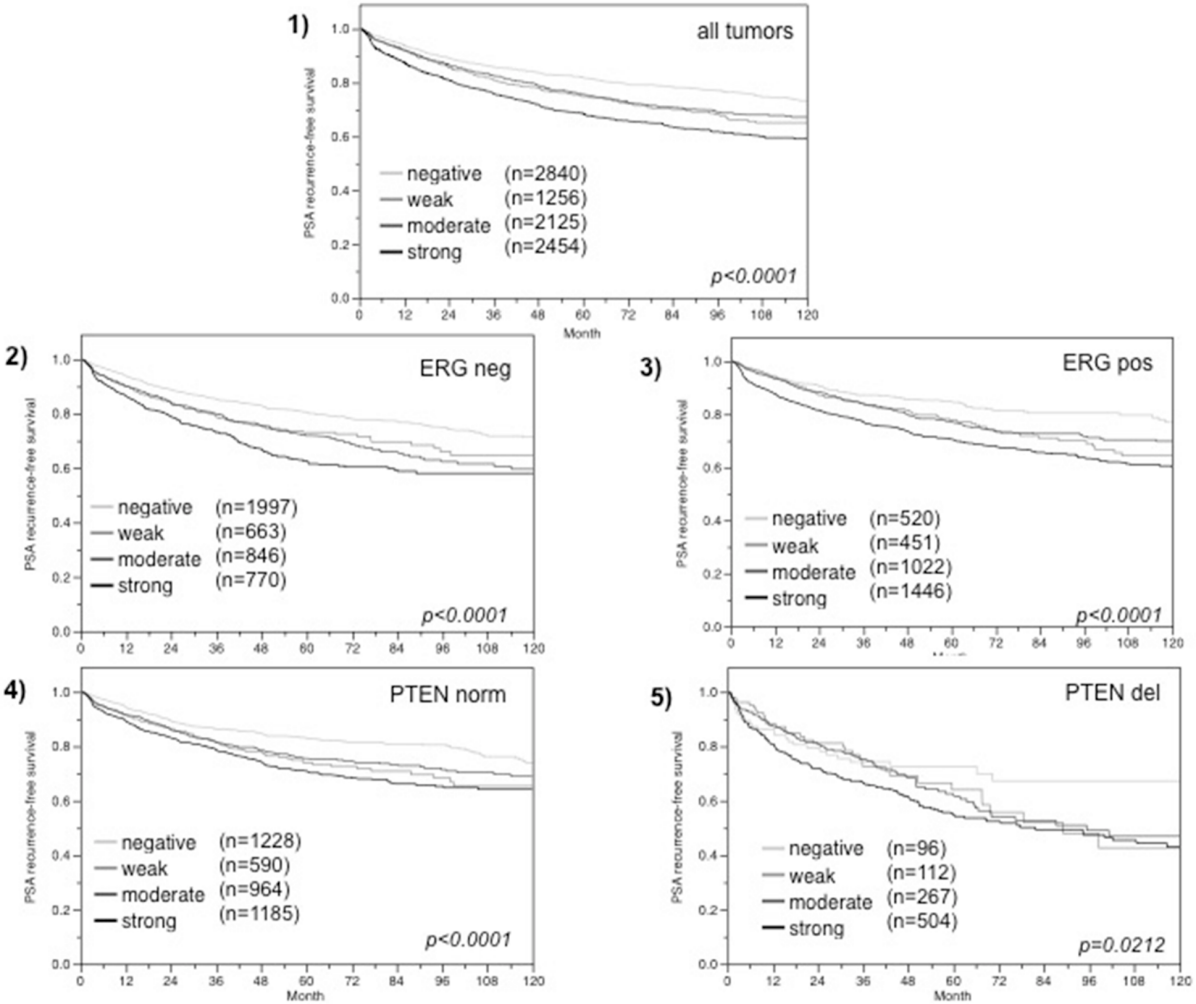
Association between FAM13C expression and biochemical recurrence in (**1**) all cancers, (**2**) ERG fusion negative cancers, (**3**) ERG fusion positive cancers, and (**4**) tumors without (PTEN norm) and (**5**) tumors with (PTEN del) *PTEN* deletion.

**Figure 7:**
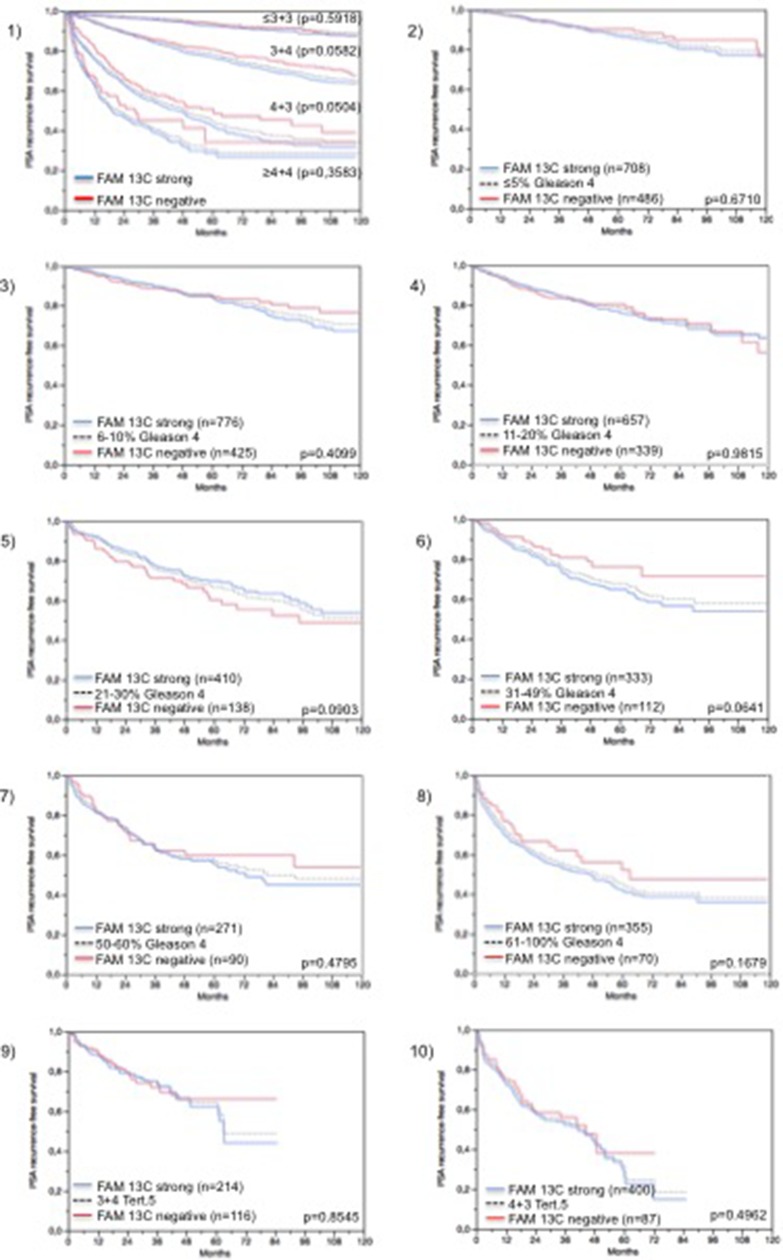
Prognostic impact of FAM13C expression in subsets of cancers defined by the Gleason score (**1**) Impact of negative (red line) and strongly positive (blue line) FAM13C expression as compared to the classical Gleason score categories (indicated by black dotted lines). 2–10) Impact of negative (blue line) and strongly positive (red line) FAM13C expression as compared to the quantitative Gleason score categories (black dotted line) defined by subsets of cancers with 1) ≤ 5% Gleason 4 patterns, (**2**) 6–10% Gleason 4 patterns, (**3**) 11–20% Gleason 4 patterns, (**4**) 21–30% Gleason 4 patterns, (**5**) 31–49% Gleason 4 patterns, (**6**) 50–60% Gleason 4 patterns, and (**7**) 61–100% Gleason 4 patterns. (**9**–**10**) Impact of negative (red line) and strongly positive (blue line) FAM13C expression in cancers with a tertiary Gleason 5 pattern, including 9) 3 + 4 tertiary grade 5 and 10) 4 + 3 tertiary grade 5.

### Multivariate analysis

Four multivariate analyses were performed evaluating the clinical relevance of FAM13C expression in different scenarios (Table 3). No 1 was utilizing all postoperatively available parameters including pathological tumor stage, pathological lymph node status (pN), surgical margin status, preoperative PSA value and pathological Gleason grade (classical and quantitative) obtained after the morphological evaluation of the entire resected prostate. Scenario 2 was utilizing all postoperatively available parameters with exception of nodal status. The rational for this approach was that the indication and extent of lymph node dissection is not standardized in the surgical therapy of prostate cancer and that excluding pN in multivariate analysis can markedly increase case numbers. Two additional scenarios had the purpose to model the preoperative situation. Scenario 3 included FAM13C expression, preoperative PSA, clinical tumor stage (cT stage) and Gleason grade obtained on the prostatectomy specimen. Since postoperative determination of a tumors Gleason grade is “better” than the preoperatively determined Gleason grade (subjected to sampling errors and consequently under-grading in more than one third of cases [17]), another multivariate analysis was added. In scenario 4, the preoperative Gleason grade obtained on the original biopsy was combined with preoperative PSA, cT stage and FAM13C expression. All these classical scenarios suggest a strong evidence of FAM13C expression levels to represent an independent predictor of prognosis (Table 3). For scenario 1–3 additional multivariate analyses were performed in which the classical Gleason grade was replaced by quantitative Gleason grade representation. Here again, FAM13C expression was an independent predictor of prognosis, even though with somewhat weaker *p*-values (Table 3; *p*-values in brackets).

**Table 3:** Multivariate analysis including FAM13C expression status

Scenario	*n* analyzable	*p* -value							FAM13C-Expression
		preop. PSA-Level	pT Stage	cT Stage	Gleason-grade prostatectomy	Gleason grade biopsy	N-Stage	R-Status	
1	5,354	*< 0.0001*	*< 0.0001*	*-*	*< 0.0001*	*-*	*< 0.0001*	*0.002*	*0.0275*
2	8,469	*< 0.0001*	*< 0.0001*	*-*	*< 0.0001*	*-*	*-*	*< 0.0001*	*0.0023*
3	8,350	*< 0.0001*	*-*	*< 0.0001*	*< 0.0001*	*-*	*-*	*-*	*< 0.0001*
4	8,239	*< 0.0001*	*-*	*< 0.0001*	*-*	*< 0.0001*	*-*	*-*	*< 0.0001*

*p* values in brackets indicate that the quantitative Gleason was used instead of the classical Gleason for multivariate modeling.

## DISCUSSION

The results of our study show that FAM13C overexpression is a strong predictor of poor clinical outcome in prostate cancer, and that its prognostic impact is independent of established pathological and clinical parameters.

Although data on FAM13C expression have never been published in prostate cancer, the gene is a component of an expression signature, which is currently proposed for routine application as a commercially available prognostic test in prostate cancer [6]. Our immunohistochemical study on 9,633 prostate cancers strongly supports a relevant role of FAM13C in this disease. Nuclear FAM13C staining was found at different levels in about two thirds of the cancers analyzed in our study, including moderate to strong expression in about 50% of tumors. Given that FAM13C staining was regularly found to be weak to moderate in normal prostate epithelium, these findings suggest that FAM13C becomes up regulated during tumor development and/or progression in a relevant fraction of prostate tumors. This was also supported by our findings that strong FAM13C expression continuously increased from benign prostate lesions (BPH and PIN) to high Gleason grade cancers, lymph node metastasis and hormone refractory cancers. Data from other cancer types seem to suggest that FAM13C upregulation can occur in malignant tumors. FAM13C upregulation has been described in gliomas, liver cancers, and lymphomas as compared to their corresponding normal tissues [18]. The strong association of high FAM13C expression with adverse tumor features, including advanced stage, high Gleason grade, nodal status and PSA recurrence argues for a practical relevance of FAM13C measurement for prognosis assessment.

The prognostic impact of FAM13C expression was independent of established prognostic features, both in preoperative and in postoperative scenarios. This highlights the potential applicability of FAM13C measurement – either alone or in combination with other factors – for a better assessment of prostate cancer aggressiveness in clinical practice. However, given the ubiquitous nature of FAM13C expression, such a potential routine test will require a diagnostic threshold for FAM13C overexpression that needs to be defined. The Gleason Grade is the strongest established prognostic parameter in prostate cancer. Based on the large cohort of prostate cancers available at our institution, we had earlier shown, that Gleason Grade information can also be used as a continuous rather than a categorical variable. Both in biopsies and in prostatectomy samples, prostate cancer prognosis continuously deteriorates with increasing percentage of unfavorable Gleason pattern found in a cancer (quantitative Gleason Grade) [19]. That FAM13C expression continuously increases with the percentage of Gleason 4 fractions in our patients further emphasizes the strong link of FAM13C expression with prostate cancer aggressiveness. The lack of an unequivocal prognostic impact of FAM13C expression in subgroups defined by a comparable quantitative Gleason grade also demonstrates how difficult it is – even for very good biomarkers – to surpass morphological parameters of malignancy.

In earlier studies, using the same large prostate cancer cohort we had described other very strong and often independent prognostic features such as for example ß3-tubulin [20], CD57 [21], DAXX [22], HOXB13 [23], KPNA2 [24], RBM3 [25], mTOR [26], p62 [27], and TYMS [28], that might also be worth testing in multiparametric prognostic kits. It is noteworthy, however, that FAM13C as well as many other prognostic features such as HOXB13 [23], CD147 [29], FOXP2 [30], CD151 [31], c-MET [32] or p27 [33] are not only expressed in cancer cells but also in normal prostatic epithelium as well as in basal (FOXP2, c-MET), inflammatory (CD117, FOXP3) [34, 35], endothelial (CD151), neuronal (ß3-tubulin) [20], or stromal cells (FAM13C). It is currently unknown to what extent this obvious expression in non neoplastic cells limits the applicability of RNA based prognosis testing, as is currently proposed by commercial vendors [6, 36, 37].

In this study, we analyzed a protein with largely unknown function. The immunohistochemical analysis revealed that the FAM13C protein is localized in the cell nucleus, which would be compatible with a role in DNA synthesis and repair, expression control, chromatin remodeling, or maintenance of nuclear architecture. That FAM13C expression was also found in cancer cells and also-typically at lower levels-in non-neoplastic tissues including luminal, basal and stroma cells further argues for a general metabolic function. The extensive molecular database attached to our tumors enabled us to draw some further “*in silico*” conclusions on potential FAM13C functions. The marked association of FAM13C expression and cell proliferation found in our study might for example support a role of FAM13C in growth regulation or cell homeostasis. A role in regulating cell proliferation has also been suggested for other FAMs. FAM83B was shown to trigger cell growths by activating EGFR/RAS/MAPK signaling in human mammary epithelial (HME1) cells [38, 39] and FAM83D enhanced cell proliferation in MCF10A breast cells [38, 40], while FAM176A induced growth arrest in H1299 non-small cell lung cancer cells [41] and FAM43B suppressed cell proliferation in HCC cell lines [42]. Given that all FAM members show a high degree of sequence homology, it might be possible that they share functional patterns.

The comparison of FAM13C with established molecular features in prostate cancer demonstrated that increased FAM13C expression is strongly associated with the subset of tumors harboring the *TMPRSS2*:*ERG* gene fusion. More than half of all prostate cancers carry this gene fusion which links the androgen-regulated *TMPRSS2* gene with the transcription factor *ERG* [8, 43] resulting in an androgen-dependent overexpression of the *ERG* transcription factor [44]. The strong link between FAM13C and ERG expression fits well to earlier work suggesting that FAM13C is a target gene of AR and ERG, given that the FAM13C promoter carries both binding sites for AR and ERG in close proximity [45]. Similar associations with ERG have also been found for other FAM members, including FAM77C [46] and FAM13A [14] that are up regulated in the presence of ERG, or for FAM111B [47], FAM3B and FAM124B [14] that are down-regulated in ERG positive cancers.

Our “*in silico*” functional analysis further identified a striking association of FAM13C positivity with multiple chromosomal deletions, particularly in ERG negative cancers. That these associations were markedly reduced in ERG positive cancers may be explained by the fact that the markedly higher FAM13C expression levels in ERG positive than in ERG negative cancers makes it more difficult to see further differences in expression under the selected experimental conditions. This was most obviously the case for *PTEN* but also seen for 5q and 6q. That FAM13C up regulation is linked to a higher prevalence of all these deletions suggests a possible impact of FAM13C on mechanisms regulating genomic integrity. In addition, the particularly strong and ERG-independent association with *PTEN* deletions argues for a functional interaction between both genes. PTEN is a multifunctional lipid phosphatase that negatively regulates the phosphatidylinositol (PI)-3 kinase/AKT growth pathway [48] but is also involved in DNA repair [49]. It is, thus, tempting to speculate, that FAM13C might interact with *PTEN* deletion both in growth regulation and maintenance of genome stability. A joint role in growth control is supported by our observation that FAM13C overexpression was linked to increased cell proliferation even in *PTEN* deleted cancers. We have previously found similar associations between *PTEN* deletion and other proteins that are known to functionally interfere with PTEN signaling, including p53 [50] and mTOR [26], in our TMA. However, functional analyses are required to elucidate the role of FAM13C in *PTEN* deleted cancers.

In summary, the results of our study show that overexpression of FAM13C – a gene of largely unknown function-is a strong and independent prognostic feature in prostate cancer. Comparison with a plethora of molecular data available from our patient cohort suggests AR dependency of FAM13C and possible roles in controlling cell cycle and genetic integrity.

## MATERIALS AND METHODS

### Patients

Radical prostatectomy specimens were available from 12,427 patients, undergoing surgery between 1992 and 2012 at the Department of Urology and the Martini Clinic at the University Medical Center Hamburg-Eppendorf. Histo-pathological data was retrieved from the patient files, including tumor stage, Gleason grade, nodal stage and stage of the resection margin. In addition to the classical Gleason categories, “quantitative” Gleason grading was performed as described before [19]. In brief, for every prostatectomy specimen, the percentages of Gleason 3, 4, and 5 patterns were estimated in cancerous tissues during the regular process of Gleason grading. Gleason 3 + 4 and 4 + 3 cancers were subdivided according to their percentage of Gleason 4. For practical use, we subdivided the 3 + 4 and 4 + 3 cancers in 8 subgroups: 3 + 4 ≤ 5% Gleason 4, 3 + 4 6–10%, 3 + 4 11–20%, 3 + 4 21–30%, 3 + 4 31–49%, 4 + 3 50–60%, 4 + 3 61–80% and 4 + 3 > 80% Gleason 4. In addition, separate groups were defined by the presence of a tertiary Gleason 5 pattern, including 3 + 4 Tert.5 and 4 + 3 Tert. 5. Follow-up data were available for a total of 12,344 patients with a median follow-up of 36 months (range: 1 to 241 months; Table 4). Prostate specific antigen (PSA) values were measured following surgery and PSA recurrence was defined as a postoperative PSA of 0.2 ng/ml and increasing at first of appearance. All prostate specimens were analyzed according to a standard procedure, including a complete embedding of the entire prostate for histological analysis [51]. The TMA manufacturing process was described earlier in detail [52]. In short, one 0.6mm core was taken from a representative tissue block from each patient. The tissues were distributed among 27 TMA blocks, each containing 144 to 522 tumor samples. For internal controls, each TMA block also contained various control tissues, including normal prostate tissue. The molecular database attached to this TMA contained results on ERG expression in 10,678 [8] und Minner, *ERG* break apart FISH analysis in 7,099 (expanded from [53] and deletion status of 5q21 (*CHD1*) in 7,932 (expanded from [12]), 6q15 (*MAP3K7*) in 6,069 (expanded from [11]), *PTEN* (10q23) in 6,704 (expanded from [9) and 3p13 (*FOXP1*) in 7,081 (expanded from [10]) cancers. In addition, a second small “prostate cancer progression” TMA was analyzed containing samples from 100 benign prostate hyperplasias (BPH), and 50 samples each form prostatic intraepithelial neoplasias (PIN), high Gleason grade cancers (Gleason 8–9), lymph node metastasis, and hormone refractory cancers. The usage of archived diagnostic left-over tissues for manufacturing of tissue microarrays and their analysis for research purposes as well as patient data analysis has been approved by local laws (HmbKHG, §12,1) and by the local ethics committee (Ethics commission Hamburg, WF-049/09 and PV3652). All work has been carried out in compliance with the Helsinki Declaration.

**Table 4:** Composition of the prognosis tissue microarray containing 12,427 prostate cancer specimens

	No. of patients (%)	
	Study cohort on TMA	Biochemical relapse among categories
	(***n*** **= 12,427)**	
**Follow-up (mo)**		
*n*	11,665 (93.9%)	2,769 (23.7%)
Mean	48.9	-
Median	36.4	-
**Age (y)**		
≤ 50	334 (2.7%)	81 (24.3%)
51–59	3,061 (24.8%)	705 (23%)
60–69	7,188 (58.2%)	1,610 (22.4%)
≥ 70	1,761 (14.3%)	370 (21%)
**Pretreatment PSA (ng/ml)**		
<4	1,585 (12.9%)	242 (15.3%)
4–10	7,480 (60.9%)	1,355 (18.1%)
10–20	2,412 (19.6%)	737 (30.6%)
> 20	812 (6.6%)	397 (48.9%)
**pT stage (AJCC 2002)**		
pT2	8,187 (66.2%)	1,095 (13.4%)
pT3a	2,660 (21.5%)	817 (30.7%)
pT3b	1,465 (11.8%)	796 (54.3%)
pT4	63 (0.5%)	51 (81%)
**Gleason grade**		
≤ 3 + 3	2,983 (24.1%)	368 (12.3%)
3 + 4	6,945 (56.2%)	1,289 (18.6%)
4 + 3	1,848 (15%)	788 (42.6%)
≥ 4 + 4	584 (4.7%)	311 (53.3%)
**pN stage**		
pN0	6,970 (91%)	1,636 (23.5%)
pN+	693 (9%)	393 (56.7%)
**Surgical margin**		
Negative	9,990 (81.9%)	1,848 (18.5%)
Positive	2,211 (18.1%)	853 (38.6%)

### Immunohistochemistry

Freshly cut TMA sections of the 12,427 samples TMA were immunostained on one day and in one experiment. The small “prostate progression TMA” was analyzed later using a different batch of the FAM13C antibody. Slides were deparaffinized and exposed to heat-induced antigen retrieval for 5 minutes in an autoclave at 121°C in pH 7.8 Tris-EDTA-Citrate buffer. Primary antibody specific for FAM13C (rabbit polyclonal antibody, Sigma-Aldrich, St. Louis, MO; cat#HPA037888; dilution 1:150) was applied at 37°C for 60 minutes. A preabsorption control assay using purified FAM13C protein (APRET80276, Sigma-Aldrich) in 50-fold excess relative to the primary antibody was performed to prove specificity of the antibody (Supplementary Figure 1). Specificity of the antibody for its target protein was also demonstrated in the Human Protein Atlas project (www.proteinatlas.org, [54], query FAM13C) by protein array analysis and by the antibody manufacturer using western blotting. Bound antibody was then visualized using the EnVision Kit (Dako, Glostrup, Denmark) according to the manufacturer´s directions. FAM13C stained the tumor cell nuclei in all (100%) cells of a tissue spot. Staining intensity of all cases was thus semiquantitatively assessed in four categories: negative, weak, moderate and strong. The percentage of positive tumor cells (typically 100% for this staining) was not seperately recorded.

### Statistics

For statistical analysis, the JMP 9.0 software (SAS Institute Inc., NC, USA) was used. Contingency tables were calculated to study association between FAM13C expression and clinico-pathological variable, and the Chi-square (Likelihood) test was used to find significant relationships. Kaplan Meier curves were generated for PSA recurrence free survival. The log-Rank test was applied to test the significance of differences between stratified survival functions. Cox proportional hazards regression analysis was performed to test the statistical independence and significance between pathological, molecular, and clinical variables.

## SUPPLEMENTARY MATERIALS FIGURES AND TABLES



## Abbreviations

cT: clinical stage
Li: labeling index
PSA: prostate specific antigen
pT: pathological stage
pN: nodal stage
R: surgical margin
TMA: tissue micro array

## References

[1] Torre LA, Bray F, Siegel RL, Ferlay J, Lortet-Tieulent J, Jemal A (2015). Global cancer statistics, 2012. CA Cancer J Clin.

[2] Wilt TJ, Brawer MK, Jones KM, Barry MJ, Aronson WJ, Fox S, Gingrich JR, Wei JT, Gilhooly P, Grob BM, Nsouli I, Iyer P, Cartagena R (2012). Radical prostatectomy versus observation for localized prostate cancer. N Engl J Med.

[3] Thompson IM, Tangen CM (2012). Prostate cancer—uncertainty and a way forward. N Engl J Med.

[4] Cohen M, Reichenstein M, Everts-van der Wind A, Heon-Lee J, Shani M, Lewin HA, Weller JI, Ron M, Seroussi E (2004). Cloning and characterization of FAM13A1—a gene near a milk protein QTL on BTA6: evidence for population-wide linkage disequilibrium in Israeli Holsteins. Genomics.

[5] Gasi Tandefelt D, Boormans JL, van der Korput HA, Jenster GW, Trapman J (2013). A 36-gene signature predicts clinical progression in a subgroup of ERG-positive prostate cancers. Eur Urol.

[6] Knezevic D, Goddard AD, Natraj N, Cherbavaz DB, Clark-Langone KM, Snable J, Watson D, Falzarano SM, Magi-Galluzzi C, Klein EA, Quale C (2013). Analytical validation of the Oncotype DX prostate cancer assay - a clinical RT-PCR assay optimized for prostate needle biopsies. BMC Genomics.

[7] Minner S, Enodien M, Sirma H, Luebke AM, Krohn A, Mayer PS, Simon R, Tennstedt P, Muller J, Scholz L, Brase JC, Liu AY, Schluter H (2011). ERG status is unrelated to PSA recurrence in radically operated prostate cancer in the absence of antihormonal therapy. Clin Cancer Res.

[8] Weischenfeldt J, Simon R, Feuerbach L, Schlangen K, Weichenhan D, Minner S, Wuttig D, Warnatz HJ, Stehr H, Rausch T, Jager N, Gu L, Bogatyrova O (2013). Integrative genomic analyses reveal an androgen-driven somatic alteration landscape in early-onset prostate cancer. Cancer cell.

[9] Krohn A, Diedler T, Burkhardt L, Mayer PS, De Silva C, Meyer-Kornblum M, Kotschau D, Tennstedt P, Huang J, Gerhauser C, Mader M, Kurtz S, Sirma H (2012). Genomic deletion of PTEN is associated with tumor progression and early PSA recurrence in ERG fusion-positive and fusion-negative prostate cancer. Am J Pathol.

[10] Krohn A, Seidel A, Burkhardt L, Bachmann F, Mader M, Grupp K, Eichenauer T, Becker A, Adam M, Graefen M, Huland H, Kurtz S, Steurer S (2013). Recurrent deletion of 3p13 targets multiple tumour suppressor genes and defines a distinct subgroup of aggressive ERG fusion-positive prostate cancers. J Pathol.

[11] Kluth M, Hesse J, Heinl A, Krohn A, Steurer S, Sirma H, Simon R, Mayer PS, Schumacher U, Grupp K, Izbicki JR, Pantel K, Dikomey E (2013). Genomic deletion of MAP3K7 at 6q12–22 is associated with early PSA recurrence in prostate cancer and absence of TMPRSS2: ERG fusions. Mod Pathol.

[12] Burkhardt L, Fuchs S, Krohn A, Masser S, Mader M, Kluth M, Bachmann F, Huland H, Steuber T, Graefen M, Schlomm T, Minner S, Sauter G (2013). CHD1 is a 5q21 tumor suppressor required for ERG rearrangement in prostate cancer. Cancer Res.

[13] Barbieri CE, Baca SC, Lawrence MS, Demichelis F, Blattner M, Theurillat JP, White TA, Stojanov P, Van Allen E, Stransky N, Nickerson E, Chae SS, Boysen G (2012). Exome sequencing identifies recurrent SPOP, FOXA1 and MED12 mutations in prostate cancer. Nat Genet.

[14] Taylor BS, Schultz N, Hieronymus H, Gopalan A, Xiao Y, Carver BS, Arora VK, Kaushik P, Cerami E, Reva B, Antipin Y, Mitsiades N, Landers T (2010). Integrative genomic profiling of human prostate cancer. Cancer cell.

[15] Lapointe J, Li C, Giacomini CP, Salari K, Huang S, Wang P, Ferrari M, Hernandez-Boussard T, Brooks JD, Pollack JR (2007). Genomic profiling reveals alternative genetic pathways of prostate tumorigenesis. Cancer Res.

[16] Sun M, Srikantan V, Ma L, Li J, Zhang W, Petrovics G, Makarem M, Strovel JW, Horrigan SG, Augustus M, Sesterhenn IA, Moul JW, Chandrasekharappa S (2006). Characterization of frequently deleted 6q locus in prostate cancer. DNA Cell Biol.

[17] Ellis SD, Blackard B, Carpenter WR, Mishel M, Chen RC, Godley PA, Mohler JL, Bensen JT (2013). Receipt of National Comprehensive Cancer Network guideline-concordant prostate cancer care among African American and Caucasian American men in North Carolina. Cancer.

[18] Berglund L, Bjorling E, Oksvold P, Fagerberg L, Asplund A, Szigyarto CA, Persson A, Ottosson J, Wernerus H, Nilsson P, Lundberg E, Sivertsson A, Navani S (2008). A genecentric Human Protein Atlas for expression profiles based on antibodies. Mol Cell Proteomics.

[19] Sauter G, Steurer S, Clauditz TS, Krech T, Wittmer C, Lutz F, Lennartz M, Janssen T, Hakimi N, Simon R, von Petersdorff-Campen M, Jacobsen F, von Loga K (2016). Clinical Utility of Quantitative Gleason Grading in Prostate Biopsies and Prostatectomy Specimens. Eur Urol.

[20] Tsourlakis MC, Weigand P, Grupp K, Kluth M, Steurer S, Schlomm T, Graefen M, Huland H, Salomon G, Steuber T, Wilczak W, Sirma H, Simon R (2014). betaIII-tubulin overexpression is an independent predictor of prostate cancer progression tightly linked to ERG fusion status and PTEN deletion. Am J Pathol.

[21] Wangerin H, Kristiansen G, Schlomm T, Stephan C, Gunia S, Zimpfer A, Weichert W, Sauter G, Erbersdobler A (2014). CD57 expression in incidental, clinically manifest, and metastatic carcinoma of the prostate. Biomed Res Int.

[22] Tsourlakis MC, Schoop M, Plass C, Huland H, Graefen M, Steuber T, Schlomm T, Simon R, Sauter G, Sirma H, Minner S (2013). Overexpression of the chromatin remodeler death-domain-associated protein in prostate cancer is an independent predictor of early prostate-specific antigen recurrence. Hum Pathol.

[23] Zabalza CV, Adam M, Burdelski C, Wilczak W, Wittmer C, Kraft S, Krech T, Steurer S, Koop C, Hube-Magg C, Graefen M, Heinzer H, Minner S (2015). HOXB13 overexpression is an independent predictor of early PSA recurrence in prostate cancer treated by radical prostatectomy. Oncotarget.

[24] Grupp K, Boumesli R, Tsourlakis MC, Koop C, Wilczak W, Adam M, Sauter G, Simon R, Izbicki JR, Graefen M, Huland H, Steurer S, Schlomm T (2014). The prognostic impact of high Nijmegen breakage syndrome (NBS1) gene expression in ERG negative prostate cancers lacking PTEN deletion is driven by KPNA2 expression. Int J Cancer.

[25] Grupp K, Wilking J, Prien K, Hube-Magg C, Sirma H, Simon R, Steurer S, Budaus L, Haese A, Izbicki J, Sauter G, Minner S, Schlomm T (2014). High RNA-binding motif protein 3 expression is an independent prognostic marker in operated prostate cancer and tightly linked to ERG activation and PTEN deletions. Eur J Cancer.

[26] Muller J, Ehlers A, Burkhardt L, Sirma H, Steuber T, Graefen M, Sauter G, Minner S, Simon R, Schlomm T, Michl U (2013). Loss of pSer2448-mTOR expression is linked to adverse prognosis and tumor progression in ERG-fusion-positive cancers. Int J Cancer.

[27] Burdelski C, Reiswig V, Hube-Magg C, Kluth M, Minner S, Koop C, Graefen M, Heinzer H, Tsourlakis MC, Wittmer C, Huland H, Huehne-Simon J, Schlomm T (2015). Cytoplasmic accumulation of Sequestosome 1 (p62) is a predictor of biochemical recurrence, rapid tumor cell proliferation and genomic instability in prostate cancer. Clin Cancer Res.

[28] Burdelski C, Strauss C, Tsourlakis MC, Kluth M, Hube-Magg C, Melling N, Lebok P, Minner S, Koop C, Graefen M, Heinzer H, Wittmer C, Krech T (2015). Overexpression of thymidylate synthase (TYMS) is associated with aggressive tumor features and early PSA recurrence in prostate cancer. Oncotarget.

[29] Grupp K, Hohne TS, Prien K, Hube-Magg C, Tsourlakis MC, Sirma H, Pham T, Heinzer H, Graefen M, Michl U, Simon R, Wilczak W, Izbicki J (2013). Reduced CD147 expression is linked to ERG fusion-positive prostate cancers but lacks substantial impact on PSA recurrence in patients treated by radical prostatectomy. Exp Mol Pathol.

[30] Stumm L, Burkhardt L, Steurer S, Simon R, Adam M, Becker A, Sauter G, Minner S, Schlomm T, Sirma H, Michl U (2013). Strong expression of the neuronal transcription factor FOXP2 is linked to an increased risk of early PSA recurrence in ERG fusion-negative cancers. J Clin Pathol.

[31] Minner S, De Silva C, Rink M, Dahlem R, Chun F, Fisch M, Hoppner W, Wagner W, Bokemeyer C, Terracciano L, Simon R, Sauter G, Wilczak W (2012). Reduced CD151 expression is related to advanced tumour stage in urothelial bladder cancer. Pathology.

[32] Jacobsen F, Ashtiani SN, Tennstedt P, Heinzer H, Simon R, Sauter G, Sirma H, Tsourlakis MC, Minner S, Schlomm T, Michl U (2013). High c-MET expression is frequent but not associated with early PSA recurrence in prostate cancer. Exp Ther Med.

[33] Sirma H, Broemel M, Stumm L, Tsourlakis T, Steurer S, Tennstedt P, Salomon G, Michl U, Haese A, Simon R, Sauter G, Schlomm T, Minner S (2013). Loss of CDKN1B/p27Kip1 expression is associated with ERG fusion-negative prostate cancer, but is unrelated to patient prognosis. Oncol Lett.

[34] Fleischmann A, Schlomm T, Kollermann J, Sekulic N, Huland H, Mirlacher M, Sauter G, Simon R, Erbersdobler A (2009). Immunological microenvironment in prostate cancer: High mast cell densities are associated with favorable tumor characteristics and good prognosis. Prostate.

[35] Flammiger A, Weisbach L, Huland H, Tennstedt P, Simon R, Minner S, Bokemeyer C, Sauter G, Schlomm T, Trepel M (2013). High tissue density of FOXP3+ T cells is associated with clinical outcome in prostate cancer. Eur J Cancer.

[36] Cooperberg MR, Simko JP, Cowan JE, Reid JE, Djalilvand A, Bhatnagar S, Gutin A, Lanchbury JS, Swanson GP, Stone S, Carroll PR (2013). Validation of a cell-cycle progression gene panel to improve risk stratification in a contemporary prostatectomy cohort. J Clin Oncol.

[37] Erho N, Crisan A, Vergara IA, Mitra AP, Ghadessi M, Buerki C, Bergstralh EJ, Kollmeyer T, Fink S, Haddad Z, Zimmermann B, Sierocinski T, Ballman KV (2013). Discovery and validation of a prostate cancer genomic classifier that predicts early metastasis following radical prostatectomy. PloS one.

[38] Cipriano R, Graham J, Miskimen KL, Bryson BL, Bruntz RC, Scott SA, Brown HA, Stark GR, Jackson MW (2012). FAM83B mediates EGFR- and RAS-driven oncogenic transformation. J Clin Invest.

[39] Cipriano R, Bryson BL, Miskimen KL, Bartel CA, Hernandez-Sanchez W, Bruntz RC, Scott SA, Lindsley CW, Brown HA, Jackson MW (2014). Hyperactivation of EGFR and downstream effector phospholipase D1 by oncogenic FAM83B. Oncogene.

[40] Wang Z, Liu Y, Zhang P, Zhang W, Wang W, Curr K, Wei G, Mao JH (2013). FAM83D promotes cell proliferation and motility by downregulating tumor suppressor gene FBXW7. Oncotarget.

[41] Xie H, Hu J, Pan H, Lou Y, Lv P, Chen Y (2014). Adenovirus vector-mediated FAM176A overexpression induces cell death in human H1299 non-small cell lung cancer cells. BMB Rep.

[42] Xu X, Liu RF, Wan BB, Xing WM, Huang J, Han ZG (2011). Expression of a novel gene FAM43B repressing cell proliferation is regulated by DNA methylation in hepatocellular carcinoma cell lines. Molecular and cellular biochemistry.

[43] Tomlins SA, Rhodes DR, Perner S, Dhanasekaran SM, Mehra R, Sun XW, Varambally S, Cao X, Tchinda J, Kuefer R, Lee C, Montie JE, Shah RB (2005). Recurrent fusion of TMPRSS2 and ETS transcription factor genes in prostate cancer. Science.

[44] Clark JP, Cooper CS (2009). ETS gene fusions in prostate cancer. Nat Rev Urol.

[45] Cai C, Wang H, He HH, Chen S, He L, Ma F, Mucci L, Wang Q, Fiore C, Sowalsky AG, Loda M, Liu XS, Brown M (2013). ERG induces androgen receptor-mediated regulation of SOX9 in prostate cancer. The Journal of clinical investigation.

[46] Jhavar S, Reid A, Clark J, Kote-Jarai Z, Christmas T, Thompson A, Woodhouse C, Ogden C, Fisher C, Corbishley C, De-Bono J, Eeles R, Brewer D (2008). Detection of TMPRSS2-ERG translocations in human prostate cancer by expression profiling using GeneChip Human Exon 1.0 ST arrays. J Mol Diagn.

[47] Brase JC, Johannes M, Mannsperger H, Falth M, Metzger J, Kacprzyk LA, Andrasiuk T, Gade S, Meister M, Sirma H, Sauter G, Simon R, Schlomm T (2011). TMPRSS2-ERG -specific transcriptional modulation is associated with prostate cancer biomarkers and TGF-beta signaling. BMC Cancer.

[48] Cantley LC, Neel BG (1999). New insights into tumor suppression: PTEN suppresses tumor formation by restraining the phosphoinositide 3-kinase/AKT pathway. Proc Natl Acad Sci USA.

[49] Misra S, Mukherjee A, Karmakar P (2014). Phosphorylation of PTEN at STT motif is associated with DNA damage response. Mutat Res.

[50] Schlomm T, Iwers L, Kirstein P, Jessen B, Kollermann J, Minner S, Passow-Drolet A, Mirlacher M, Milde-Langosch K, Graefen M, Haese A, Steuber T, Simon R (2008). Clinical significance of p53 alterations in surgically treated prostate cancers. Mod Pathol.

[51] Erbersdobler A, Isbarn H, Steiner I, Schlomm T, Chun F, Mirlacher M, Sauter G (2009). Predictive Value of Prostate-specific Antigen Expression in Prostate Cancer: A Tissue Microarray Study. Urology.

[52] Kononen J, Bubendorf L, Kallioniemi A, Barlund M, Schraml P, Leighton S, Torhorst J, Mihatsch MJ, Sauter G, Kallioniemi OP (1998). Tissue microarrays for high-throughput molecular profiling of tumor specimens. Nat Med.

[53] Minner S, Wittmer C, Graefen M, Salomon G, Steuber T, Haese A, Huland H, Bokemeyer C, Yekebas E, Dierlamm J, Balabanov S, Kilic E, Wilczak W (2011). High level PSMA expression is associated with early PSA recurrence in surgically treated prostate cancer. Prostate.

[54] Uhlen M, Fagerberg L, Hallstrom BM, Lindskog C, Oksvold P, Mardinoglu A, Sivertsson A, Kampf C, Sjostedt E, Asplund A, Olsson I, Edlund K, Lundberg E (2015). Proteomics. Tissue-based map of the human proteome. Science.

