# Supplementary material for: Family with sequence similarity 13C (FAM13C) overexpression is an independent prognostic marker in prostate cancer
Source: Oncotarget. 2017 Mar 18;8(19):31494–508. doi: 10.18632/oncotarget.16357 (PMC5458224; doi:10.18632/oncotarget.16357)
Supplement: Supplementary file 1 [file oncotarget-08-31494-s001.pdf]

Family with sequence similarity 13C (FAM13C) overexpression is an independent prognostic marker in prostate cancer

Supplementary Materials

Supplementary Table 1: Association between FAM13C immunostaining results and prostate cancer phenotype in ERG fusion negative prostate cancers

Parameter	*n* evaluable	FAM13C (%)				*p* value
		negative	weak	moderate	strong	
**All cancers**	4,750	46.5	15.6	19.9	18.0	
**Tumor stage**						
pT2	3,156	51.6	15.0	18.4	15.0	*< 0.0001*
pT3a	967	39.8	17.9	21.6	20.7	
pT3b-4	611	30.6	14.7	25.2	29.5	
**Gleason grade**						
≤ 3 + 3	986	59.7	12.1	14.9	13.3	
3 + 4	2,675	49.1	16.3	19.6	15.0	*< 0.0001*
4 + 3	797	28.9	16.9	27.1	27.1	
≥ 4 + 4	271	24.4	16.6	20.7	38.4	
**Lymph node metastasis**						
N0	2,785	44.2	17.3	20.3	18.2	*< 0.0001*
N+	278	27.3	12.2	23.7	36.7	
**Preop. PSA level (ng/ml)**						
< 4	492	40.7	14.2	20.5	24.6	
4–10	2,793	48.7	15.1	19.7	16.5	*0.0001*
10–20	1,043	45.1	17.4	20.4	17.1	
> 20	382	41.4	15.4	19.6	23.6	
**Surgical margin**						
negative	3,761	47.8	15.3	19.6	17.2	*0.0014*
positive	902	41.2	16.3	20.7	21.7	

Supplementary Table 2: Association between FAM13C immunostaining results and prostate cancer phenotype in ERG fusion positive prostate cancers

Parameter	*n* evaluable	FAM13C (%)				*p* value
		negative	weak	moderate	strong	
**All cancers**	3,812	14.7	13.2	29.9	42.2	
**Tumor stage**						
pT2	2,242	17.1	14.2	30.6	38.0	*< 0.0001*
pT3a	1,037	12.7	12.6	28.4	46.3	
pT3b-4	519	8.1	10.6	30.1	51.3	
**Gleason grade**						
≤ 3 + 3	793	18.9	12.6	32.8	35.7	
3 + 4	2,256	15.6	14.5	30.3	39.6	*< 0.0001*
4 + 3	598	7.4	10.5	25.9	56.2	
≥ 4 + 4	147	8.2	10.9	24.5	56.5	
**Lymph node metastasis**						
N0	2,220	14.1	13.3	29.3	43.3	*0.0656*
N+	245	9.4	11.0	29.4	50.2	
**Preop. PSA level (ng/ml)**						
< 4	519	14.5	14.3	28.7	42.6	
4–10	2,301	14.9	13.7	30.1	41.3	*0.508*
10–20	703	15.2	10.7	30.0	44.1	
> 20	241	11.6	14.5	29.5	44.4	
**Surgical margin**						
negative	2,972	15.4	13.8	29.8	40.9	*0.0207*
positive	771	12.1	11.8	30.7	45.4	

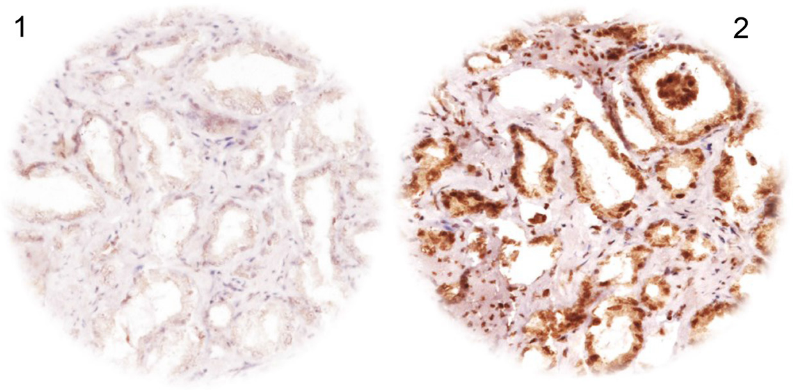

Supplementary Figure 1: Representative immuostaining pictures of specificity control of the FAM13C antibody in prostate cancer. (**1**) primary FAM13C antibody combined with preabsorption control assay and (**2**) primary FAM13C antibody alone.
